# Spatiotemporal Heterogeneity of Landscape Patterns and Ecological Risk Under Changing Human Activity in the Qilian Mountains

**DOI:** 10.1002/ece3.74497

**Published:** 2026-10-14

**Authors:** Shengji Jiang, Heng Ren, Hua Xu, Tao Zhou

**Affiliations:** ^1^ Northwest Institute of Eco‐Environment and Resources Chinese Academy of Sciences Lanzhou China; ^2^ Linze Inland River Basin Research Station Chinese Ecosystem Research Network Lanzhou China

**Keywords:** coupling, human activity intensity (HAI), landscape ecological risk (LER), landscape patterns, Qilian Mountains

## Abstract

Human activities and environmental changes can alter landscape patterns and ecological processes, with important implications for ecological risk. Taking the Qilian Mountains as a case study, we used multi‐temporal land‐use data from 2000 to 2020 to quantify human activity intensity (HAI), landscape patterns and landscape ecological risk (LER). Spatial autocorrelation and geographically weighted regression (GWR) were used to examine spatial associations between HAI and landscape patterns. HAI remained generally low but exhibited pronounced spatial heterogeneity, increasing from west to east and from higher to lower elevations. Landscape patterns were relatively stable overall, despite localized increases in fragmentation and spatial heterogeneity in aggregation and connectivity. Higher HAI was generally associated with greater fragmentation and lower aggregation and connectivity, while GWR revealed pronounced spatial non‐stationarity in these relationships. Low‐ and moderate‐low LER levels dominated, whereas moderate‐ and moderate‐high risk patches were concentrated in central‐western and high‐altitude areas, with risk increasing from southeast to northwest. The spatial relationships between HAI and LER in some high‐altitude areas indicate that ecological risk does not always correspond to the spatial distribution of human activity, highlighting the spatial complexity of ecological risk in the Qilian Mountains.

## Graphical abstract

This study investigated the spatiotemporal patterns of human activity intensity (HAI), landscape patterns, and landscape ecological risk (LER) in the Qilian Mountains from 2000 to 2020, and examined the spatial relationships between HAI and landscape patterns using spatial autocorrelation and geographically weighted regression. HAI remained generally low but spatially heterogeneous, while landscape patterns were relatively stable overall; the relationships between HAI and landscape indices showed pronounced spatial heterogeneity, and low‐ and moderate‐low‐risk areas dominated despite localized high‐risk patches in central‐western and high‐altitude areas. These findings highlight the complex and spatially heterogeneous relationships among human activity, landscape patterns, and ecological risk, providing a basis for spatially differentiated and adaptive ecosystem management in high‐altitude mountain regions.

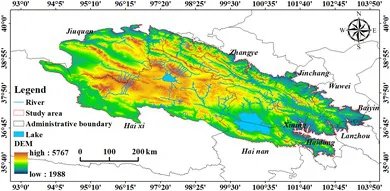

## 1 Introduction

Landscape patterns represent integrated outcomes of long‐term interactions between natural processes and human activities (Wu and Barrett 2022; Tas et al. 2018; Wang et al. 2019). Their spatial configuration and compositional characteristics reflect the distribution of land cover types and exert a profound influence on energy flows, material cycling, and ecological process stability (Liu, Zhang, et al. 2024). With the intensification of global change and human activities, landscape patterns have undergone substantial alterations in spatial arrangement and structural integrity, thereby exerting far‐reaching impacts on regional ecosystem functioning and ecological security (Keys et al. 2019; Sayles et al. 2019). Landscape pattern change has been widely recognized as a critical link between natural environmental variations and anthropogenic disturbances, thereby serving as a key entry point for elucidating ecosystem response mechanisms and assessing ecological risks (Zhang et al. 2022). Beyond natural factors, such as temperature, precipitation, and topography, anthropogenic drivers, including population distribution, socioeconomic development, and policy interventions, continuously reshape landscape structures through land use change, infrastructure construction, and resource exploitation (Huang et al. 2022). In mountainous ecosystems, where environmental conditions are harsh and ecological vulnerability is high, the impacts of human disturbances are often amplified, rendering ecosystem stability more sensitive to landscape pattern changes (Zhang et al. 2022). Consequently, systematically characterizing the spatiotemporal dynamics of human activities and elucidating their relationships with landscape pattern evolution is a fundamental prerequisite for understanding ecosystem degradation mechanisms and advancing sustainable development in mountainous regions.

Human disturbances typically manifest in multiple forms, including urban expansion, resource exploitation, agricultural utilization, and infrastructure development (Foley et al. 2005). To quantitatively characterize the integrated impacts of human activities on the ecological environment, scholars have proposed the “Human Activity Intensity” (HAI) index, which has been widely applied in ecological impact assessments and land system studies (Mao et al. 2023). Previous studies have frequently used socioeconomic indicators, such as population density and gross domestic product (GDP) to represent HAI (Xu et al. 2016; Cheng et al. 2022). However, these indicators are typically derived from administrative unit statistics, which increases the difficulty of capturing the actual spatial distribution of human activities, and are often sensitive to variations in statistical scales and methodologies employed (Zhang et al. 2015). In contrast, land use change directly reflects the ways in which human activities transform the Earth's surface and is therefore considered a more robust and spatially explicit indicator for characterizing the intensity and spatial heterogeneity of human activity (Dou et al. 2023). Adjustments in land use structures frequently cause the reorganization of landscape patches and change their spatial configuration. Thus, such adjustments exert profound effects on landscape patterns and ecological processes (Huang et al. 2022).

Although substantial progress has been made in recent years on ecological problems induced by human disturbances (Jia et al. 2020), previous studies have not fully elucidated the relationships between HAI and landscape patterns. On the one hand, human activities are often treated as auxiliary variables and analyzed qualitatively, which has led to a lack of quantitative characterizations based on spatial data. On the other hand, analyses of landscape pattern change have largely focused on a single metric or limited sets of metrics and thus have failed to systematically capture the integrated effects of human activities on landscape structure across multiple dimensions, such as fragmentation, connectivity, and diversity (Li, Wang, et al. 2021). More broadly, increasing evidence indicates that ecosystem responses to human activities are mediated by changes in land use and are strongly conditioned by spatial heterogeneity (An et al. 2024). Land‐use transformation can modify not only landscape composition and configuration but also ecosystem services, vegetation conditions, and other ecological functions, while policy interventions and socioeconomic activities may further alter these responses across space (Karadeniz et al. 2026; Wang et al. 2025). These findings highlight the need to examine human activities, land‐use change, and ecosystem responses within an integrated spatial framework rather than as isolated components. Nevertheless, relatively few studies have explicitly linked spatially quantified HAI with multidimensional changes in landscape structure and their associated ecological consequences, particularly in ecologically fragile mountain ecosystems. Consequently, developing a land‐use‐based framework for characterizing HAI and quantitatively examining its coupling with landscape patterns across multiple metrics and further evaluating the ecological implications of such landscape restructuring remain critical directions for further research.

Against this backdrop, landscape ecological risk (LER) assessments provide an important theoretical framework for understanding the integrated effects of human activities and landscape pattern changes on ecosystem stability (Jiang et al. 2023; Sayles et al. 2019). LER assessments aim to evaluate the likelihood of ecosystem degradation or destabilization under the combined influence of natural and anthropogenic disturbances and encompass multiple factors, such as climate, topography, vegetation, and human activities (Gu et al. 2025; Iqbal et al. 2026). Human activities can generate ecological responses through multiple pathways, while these responses can be evaluated from different perspectives (Lu et al. 2024). Ecosystem service assessments primarily characterize changes in ecosystem functions and the benefits that ecosystems provide to human societies, whereas habitat quality assessments focus more specifically on habitat suitability and degradation in response to environmental threats (Broquet et al. 2024). These approaches are valuable for evaluating particular dimensions of ecosystem response. For this purpose, landscape‐based LER assessment is particularly appropriate because it integrates land‐use composition, landscape disturbance, and ecological vulnerability within a spatially explicit framework (Zeng et al. 2024). It provides a consistent approach for connecting HAI‐related land‐use transformation, landscape restructuring, and their potential ecological consequences. Moreover, LER assessments emphasize the coupling between ecological processes and landscape spatial patterns, with particular attention to the spatial heterogeneity and scale effects of ecological risks (Chen et al. 2024). Therefore, LER models constructed based on land use structures not only capture differences in HAI but also reveal ecosystem sensitivity to disturbances by assigning distinct vulnerability weights to different land use types (Meng, Meng, et al. 2024). Previous studies have demonstrated the applicability of landscape‐based LER approaches across diverse regional ecological risk assessments (Gu et al. 2025; Li, Abuduwailia, et al. 2024; Li, Hu, et al. 2024).

The Qilian Mountains, located in the arid region of northwestern China, constitute a critical ecological barrier and water conservation area that sustains the Hexi Corridor and inland river basins, thus playing an irreplaceable role in ecosystem services at both regional and national scales (Liu et al. 2025). However, since the early 21st century, intensified human activities, including mineral resource exploitation, hydropower development, tourism expansion, and overgrazing, have caused pronounced disturbances to the structure and functions of local ecosystems in the Qilian Mountains (Qian et al. 2021). Coupled with the inherent ecological fragility and limited resilience of high‐altitude mountainous environments, this intensification has caused particularly prominent landscape pattern changes and ecological risks in the region. Under the combined influence of natural factors and human activities, systematically elucidating the evolution of landscape patterns, spatiotemporal dynamics of HAI, and their integrated effects on LER in the Qilian Mountains is of substantial practical significance for regional ecological conservation and management.

Building upon the above considerations, this study considered the Qilian Mountains as the study area and employed multi‐temporal land use data from 2000 to 2020 to systematically analyze the spatiotemporal evolution of landscape patterns. A land‐use‐based model for characterizing HAI was used to quantitatively depict the spatiotemporal distribution of human activities, examine the spatial associations and spatially varying relationships between HAI and landscape patterns, and comprehensively assess the spatiotemporal distribution and evolutionary characteristics of LER in the Qilian Mountains. This study aims to advance our understanding of the spatial relationships among human activities, landscape patterns, and ecological risk in high‐altitude mountainous regions, thereby providing a scientific foundation for spatially differentiated ecological management, human activity regulation, and adaptive sustainable development in the Qilian Mountains.

## 2 Materials and Methodology

### 2.1 Study Area

The Qilian Mountains are located on the northeastern edge of the Qinghai–Tibet Plateau and at the junction of northeastern Qinghai Province and western Gansu Province in China, and they form a vulnerable region that is sensitive to climate change (Figure 1). They serve as an important material‐supply area for both the Hexi Corridor and upper reaches of the Yellow River, playing an important role in China's ecosystem services (Xue et al. 2023). In addition, they stretch from the Songshan Mountains at the eastern end of the Wudang Ridge to the Dangjin Pass at the western end, border the Hexi Corridor to the north, and connect to the Qaidam Basin in the south. They span approximately 850 km from east to west and 250–400 km from north to south, with an average altitude of 4000–5000 m. Their climate is influenced primarily by topographical and geographical factors and includes highland continental, temperate grassland, and temperate semi‐arid climates, with winters that are cold and dry and summers that are cool and humid (Meng, Zhang, and Xiao 2024). The annual average temperature is below 4°C, and the annual precipitation is approximately 400 mm. The Qilian Mountains serve as an important water conservation area and are the source region of three inland rivers: the Shiyang River, the Heihe River, and the Shule River (Yang et al. 2022). The unique geographical environment and climatic conditions have fostered the establishment of ecosystems such as forests, grasslands, wetlands, and deserts.

**FIGURE 1:**
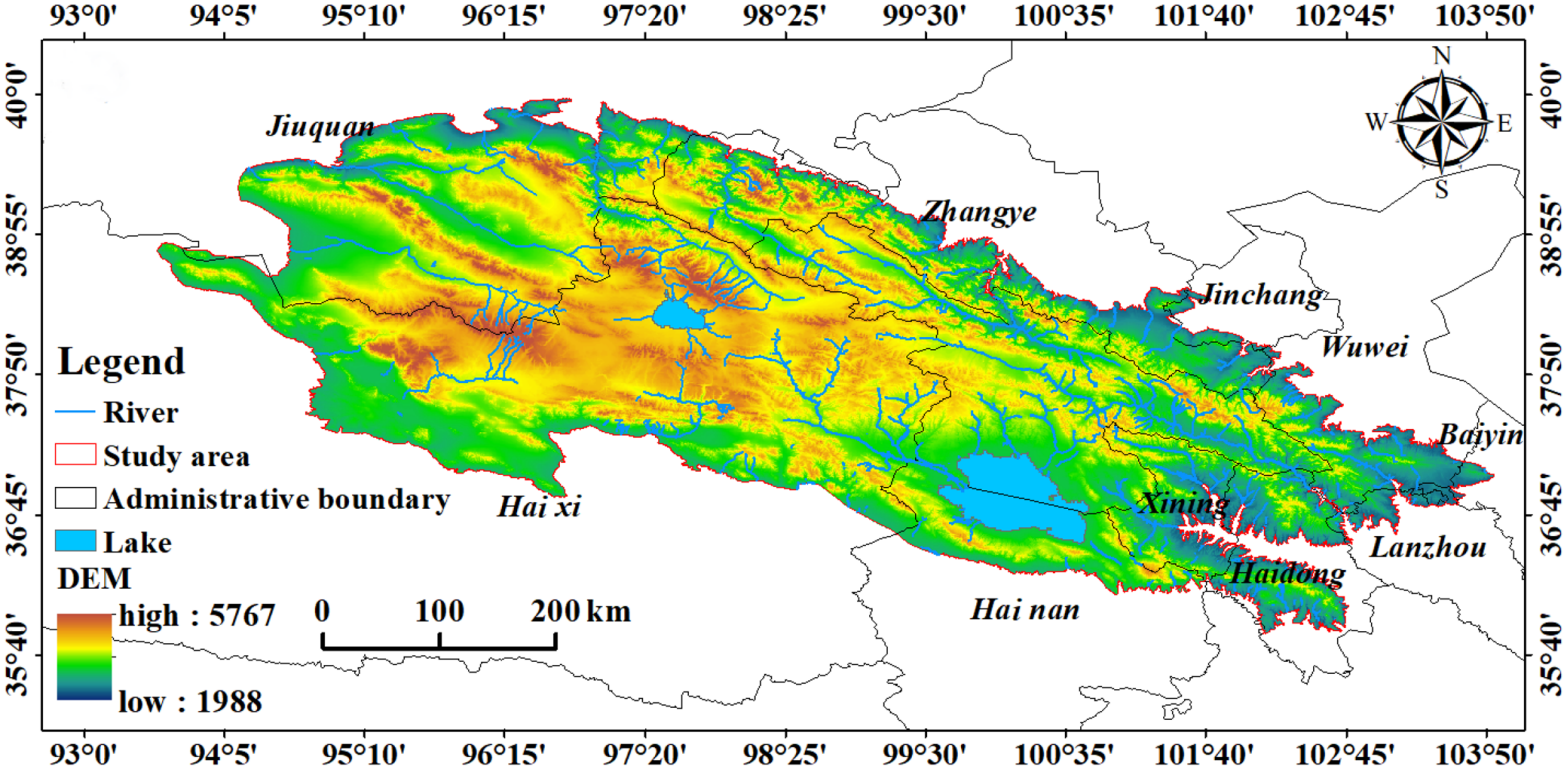
Location and overview of the study area.

### 2.2 Data Sources

Land use data from 2000 to 2020 were obtained from the China Multi‐Period Land Use Remote Sensing Monitoring Dataset of the Chinese Academy of Sciences Resource and Environmental Sciences Data Center (http://www.resdc.cn). The dataset was developed through manual visual interpretation of Landsat remote sensing imagery and provides national‐scale, multi‐period land use/land cover data at a spatial resolution of 30 m. The data were classified using a two‐tier classification system, with the first tier divided into six categories (cropland, forest land, grassland, water bodies, construction land, and unutilized land) and the second tier divided into 25 types based on the first‐tier categories. Based on China's land use/land cover data classification system and considering the actual conditions of the study area, ArcGIS was used to reclassify the land use data into six categories: cultivated land, woodland, grassland, water area, construction land, and unused land.

### 2.3 Research Methods

#### 2.3.1 Selection of Landscape Types and Indicators

Landscape indices are important statistical tools for quantitatively characterizing landscape pattern attributes (Li, Zhou, et al. 2021). By integrating information on landscape composition and spatial configuration, these indices provide comprehensive insights into the structure and evolutionary processes of ecosystems. Thus, they have been widely applied in studies on regional landscape pattern dynamics, ecological process responses, and human impact assessments (Li et al. 2023). Considering the complex topographic conditions and representative ecosystem types of the Qilian Mountains, a set of representative landscape indices was systematically selected at both the landscape and class levels to characterize landscape fragmentation, spatial aggregation, diversity patterns, and ecological connectivity in the study area (Jia et al. 2020).

At the landscape level, the patch density (PD) index, aggregation index (AI), Shannon's diversity index (SHDI), Shannon's evenness index (SHEI), contagion index (CONTAG), and patch cohesion index (COHESION) were selected. PD measures the number of patches per unit area and serves as a core indicator of landscape fragmentation. AI and CONTAG describe the degree of spatial aggregation and dispersion of patches from different perspectives and collectively reflect the overall landscape aggregation and spatial continuity. COHESION emphasizes the physical connectedness among patches of the same landscape type and thus represents structural landscape connectivity. SHDI and SHEI characterize the landscape compositional complexity and area distribution evenness among landscape types, respectively. At the class level, PD, AI, landscape shape index (LSI), and related metrics were selected for different land use/land cover types. Class‐level PD was used to evaluate the fragmentation status of specific landscape types, AI was applied to reflect their spatial aggregation characteristics, and LSI was used to quantify class‐level shape complexity, thereby revealing the boundary irregularity and spatial heterogeneity during landscape expansion processes.

#### 2.3.2 Characterization of the Human Activity Intensity

HAI in the Qilian Mountains was calculated using a land‐use‐based approach, with construction land, which is subject to intensive human socioeconomic activities, serving as the reference land‐use type. The areas of different land use types were converted into their equivalent construction land values based on the size of their conversion coefficients, and the total construction land equivalents for various land use types in the region were used to calculate the intensity of human activity in the Qilian Mountain area from 2000 to 2020 (Xu et al. 2016; Wang and Zhao 2023). HAI was then classified into four levels according to the established classification criteria: low intensity, low‐moderate intensity, moderate intensity, and moderate‐high intensity. The formula used is as follows:

$${\mathrm{HAI}}_{i}=\frac{S_{\mathrm{CLE}-i}}{S_{i}}\times 100\%$$

$$S_{\mathrm{CLE}-\mathrm{ij}}=\sum_{j=1}^{m}\left({\mathrm{SL}}_{\mathrm{ij}}\times {\mathrm{CI}}_{\mathrm{ij}}\right)$$

where *HAI*_*i*_ represents the normalized data of human activity intensity on the terrestrial surface, *S*_*CLE‐i*_ denotes the area of construction land equivalent in the ith region, *S*_*i*_ is the total land area of the *i*th region, *SL*_*ij*_ is the area of the *j*th land use type in the *i*th region, *CI*_*ij*_ is the conversion coefficient of construction land equivalent for the *j*th land use type in the *i*th region, *m* is the number of land use types in the *i*th region.

#### 2.3.3 Bivariate Spatial Autocorrelation Analysis

This study employed a bivariate local spatial autocorrelation approach to analyze the spatial association and coupling relationship between landscape patterns and HAI. By calculating the bivariate local Moran's *I*, the spatial correlation between the landscape pattern attributes within a given study unit and the HAI in its neighboring areas can be quantified. Spatial clustering types were classified into four categories: high‐high, low‐low, high‐low, and low‐high. High‐high and low‐low clusters indicated a positive local spatial correlations between landscape patterns and HAI, whereas high‐low and low‐high clusters indicated a negative spatial correlation. GeoDa software was used to analyze the spatial associations between landscape patterns and HAI in the Qilian Mountains, with Moran's *I* index employed to interpret the spatial distribution characteristics. The bivariate local Moran's *I* is calculated as follows:

$$I=\frac{n}{\sum_{i=1}^{n}\sum_{j=1}^{n}W_{\mathrm{ij}}}\times \frac{\sum_{i=1}^{n}\sum_{j=1}^{n}W_{\mathrm{ij}}\left(x_{i}-\bar{x}\right)\left(x_{j}-\bar{x}\right)}{\sum_{i=1}^{n}{\left(x_{i}-\bar{x}\right)}^{2}}$$

where *W*_*ij*_ represents the spatial weight adjacency matrix between spatial units *i* and *j* (*i, j* = 1, 2, 3,…, *n*), *x*_*i*_ and *y*_*j*_ are the observed values, *x̄* is the mean of *x*_*i*_, and the value of *I* ranges from −1 to 1.

#### 2.3.4 Geographically Weighted Regression Analysis

Geographically weighted regression (GWR) was employed to further quantify the spatially varying relationships between HAI and landscape patterns. Unlike global regression models that assume spatially constant relationships, GWR estimates location‐specific regression coefficients by assigning greater weights to observations closer to each target location, thereby allowing the magnitude and direction of relationships to vary across space (Li, Abuduwailia, et al. 2024; Li, Hu, et al. 2024; Zhu et al. 2024). For each grid cell, the mean values of HAI and the 6 landscape pattern indices (AI, COHESION, CONTAG, PD, SHDI, and SHEI) across the five observation years (2000, 2005, 2010, 2015, and 2020) were calculated to characterize their long‐term spatial relationships. Prior to GWR analysis, HAI and each landscape pattern index were standardized using *Z*‐score transformation to eliminate differences in measurement scales and facilitate comparison among the local regression coefficients.

Separate GWR models were constructed for each landscape pattern index, with the standardized landscape index as the dependent variable and standardized HAI as the explanatory variable. The local model can be expressed as:

$$y_{j}=\beta_{0}\left(u_{j},v_{j}\right)+\sum_{i=1}^{k}\beta_{i}\left(u_{j},v_{j}\right)x_{\mathrm{ij}}+\varepsilon_{j}$$

where *y*_*j*_ is the standardized landscape pattern index at location *j, x*_*ij*_ is the standardized HAI; (*u*_*j*_,*v*_*j*_) represents the spatial coordinates of location *j*; *β*_*0*_(*u*_*j*_,*v*_*j*_) is the location‐specific intercept; *β*_*i*_(*u*_*j*_,*v*_*j*_) is the local regression coefficient describing the spatially varying relationship between HAI and the corresponding landscape pattern index; and *ɛ*_*j*_ is the random error term.

#### 2.3.5 Landscape Ecological Risk Model

To assess the magnitude of LER, disturbance and vulnerability indices were used to construct an ecological risk model that accounts for landscape disturbances associated with natural conditions, human activities, as well as the internal vulnerability of different landscape types (Gu et al. 2025). The landscape ecological risk index (LERI) was calculated using the following formula:

$${\mathrm{LER}}_{k}=\sum_{i=1}^{n}\frac{S_{\mathrm{ki}}}{S_{k}}\sqrt{E_{i}\times F_{i}}$$

where *n* is the number of landscape types, *E*_*i*_ is the disturbance index for the landscape type, *F*_*i*_ is the vulnerability index for the landscape type, *S*_*ki*_ is the area of landscape type *i* in the *k*‐th risk sub‐area, and *S*_*k*_ is the total area of the *k*‐th risk sub‐area. Using the natural‐breaks classification method in ArcGIS, and considering the actual situation in the study area, the LER values of the Qilian Mountains were divided into five levels: low ecological risk area (Level I; < 0.31), low‐medium ecological risk area (Level II; 0.31–0.35), medium ecological risk area (Level III; 0.36–0.40), medium high ecological risk area (Level IV; 0.41–0.45), and high ecological risk area (Level V; > 0.45).

##### 2.3.5.1 Landscape Vulnerability Index

The landscape vulnerability index represents the ability of a landscape pattern to resist external disturbances. The lower the resistance, the greater the vulnerability and the higher the ecological risk. Building on previous research (Wang et al. 2023; Jin et al. 2019), the vulnerability levels of landscape types in the Qilian Mountains were divided into six grades: unused land (6), water area (5), cultivated land (4), grassland (3), woodland (2), and construction land (1). The vulnerability of each landscape type was then normalized, and the landscape vulnerability index (*F*_*i*_) was obtained.

##### 2.3.5.2 Landscape Sensitivity Index

The landscape sensitivity index represents the degree of sensitivity of landscape patterns in response to external disturbances. The greater the sensitivity, the higher the ecological risk. The superposition of the fragmentation degree (*C*_*i*_), separation degree (*N*_*i*_), and dominance degree (*D*_*i*_) was used to characterize landscape sensitivity indices (Li et al. 2023; Gu et al. 2025). The calculation formula is as follows:

$$E_{i}=\sum_{i=1}^{n}\left({\mathrm{aC}}_{i}+{\mathrm{bN}}_{i}+{\mathrm{cD}}_{i}\right)$$

where *a*, *b*, and *c* are the weights of *C*_*i*_, *N*_*i*_, and *D*_*i*_, respectively, with *a* + *b* + *c* = 1. According to relevant studies, *a*, *b*, and *c* were assigned values of 0.5, 0.3, and 0.2, respectively. The formulas used to calculate *C*_*i*_, *N*_*i*_, and *D*_*i*_ are as follows:

$$C_{i}=\frac{n_{i}}{A_{i}}$$

$$N_{i}=\frac{A}{2A_{i}}\sqrt{\frac{n_{i}}{A}}$$

$$D_{i}=\frac{Q_{i}+M_{i}}{4}+\frac{L_{i}}{2}$$

where *n*_*i*_ is the number of patches of landscape type *i*, *A*_*i*_ is the total area of landscape type *i*, *A* is the total landscape area, *Q*_*i*_ is the ratio of the number of quadrats in which patch *i* appears to the total number of quadrats, *M*_*i*_ is the ratio of the number of patches *i* to the total number of patches, and *L*_*i*_ is the ratio of the area of patch *i* to the total area of the quadrat.

## 3 Results and Analysis

### 3.1 Spatiotemporal Characteristics of Landscape Pattern in the Qilian Mountains

The spatial distribution of landscape patterns in the Qilian Mountains from 2000 to 2020 (Figure 2) indicated that the study area was predominantly covered by unused land and grassland, both of which occupied large contiguous areas and constituted the dominant landscape matrix. In high‐altitude mountainous regions, unused land and natural grassland were dominant and relatively homogeneous, characterized by strong spatial continuity and large contiguous patches. In contrast, mid‐ and low‐altitude valleys and piedmont areas exhibited a more heterogeneous land‐use composition, where cropland, grassland, water bodies, and construction land were interspersed. Construction land and cropland were primarily distributed in valley and riverine areas and showed relatively concentrated spatial distributions around towns and major transportation corridors.

**FIGURE 2:**
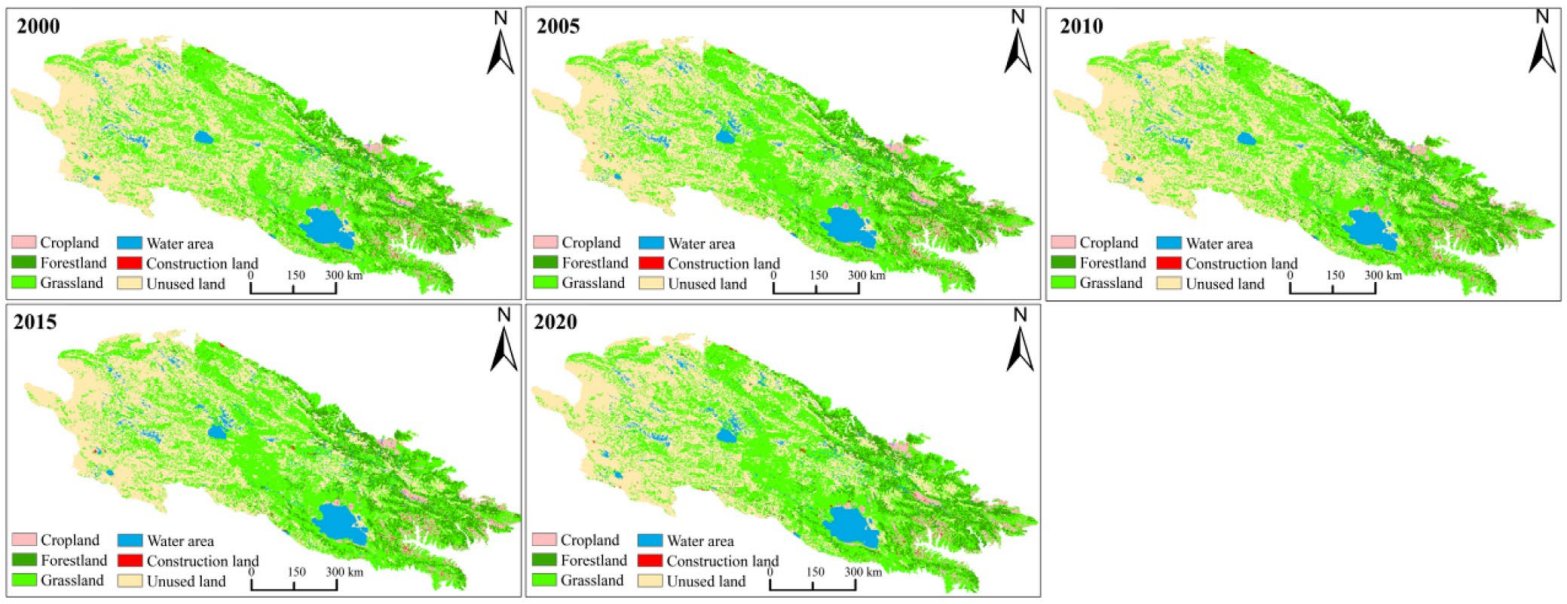
Spatial distribution of landscape patterns.

Over the period 2000–2020, the overall landscape pattern of the Qilian Mountains remained relatively stable, although localized land‐use changes were evident. The area of unused land exhibited a gradual decline, with decreases mainly occurring in some low‐altitude and valley areas, where portions were converted into grassland or cropland. Grassland area remained relatively stable during the early study period and subsequently increased, with more evident expansion after 2010. Although construction land accounted for the smallest proportion of the total area, it exhibited a notable expansion trend, especially between 2010 and 2020. Spatially, the expansion was concentrated around existing towns and along major transportation corridors, and some originally discrete construction‐land patches gradually became more spatially connected or exhibited linear extension.

### 3.2 Changes in Landscape Pattern Indices

The temporal variation of landscape pattern indices in the Qilian Mountains from 2000 to 2020 (Figure 3) indicated that PD initially increased and then decreased, suggesting that landscape fragmentation increased during the early study period but subsequently weakened. AI exhibited a gradual increasing trend, indicating a slight increase in the aggregation of patches of the same type. In contrast, CONTAG declined, indicating a gradual decrease in the overall degree of landscape contagion and an increasingly dispersed configuration among different patch types. SHDI and SHEI both showed gradual upward trends, indicating that landscape composition became increasingly diverse and that the distribution of different patch types became more even. Taken together, these indices revealed that the overall landscape pattern remained relatively stable but exhibited changes in fragmentation, aggregation, and compositional diversity during 2000–2020.

**FIGURE 3:**
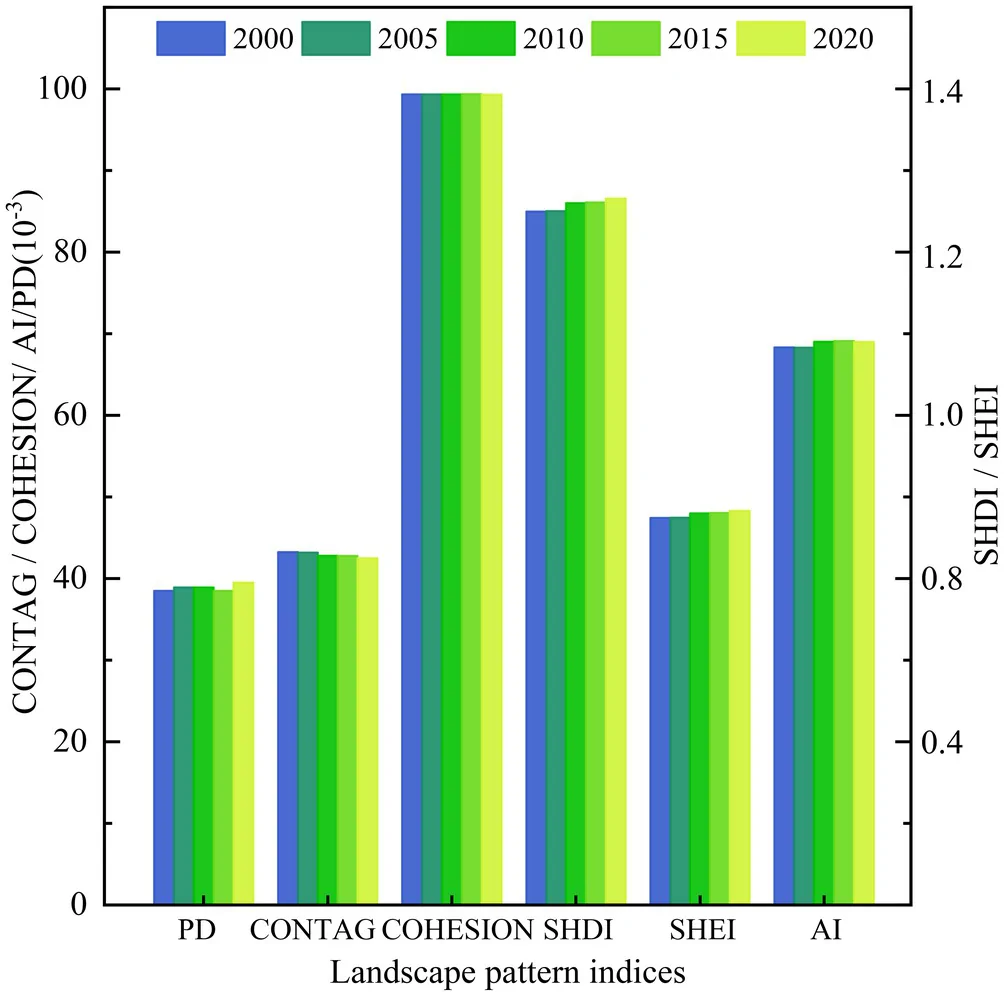
Temporal changes in landscape pattern indices.

As shown in Figure 4, landscape indices varied markedly among different landscape types. For example, grassland and unused land consistently exhibited higher PD values than the other types, indicating a relatively high density of patches within these landscape types, whereas cropland and forestland had lower PD values with limited variations, reflecting relatively stable spatial patterns. Although the PD of construction land remained generally low, it showed a gradual upward trend, reflecting a progressive increase in the density of construction land patches over time. Moreover, forestland, grassland, and water bodies consistently maintained high AI values, indicating relatively high aggregation of patches within these landscape types. In contrast, construction land had relatively low AI values but showed a slight increase over time, suggesting a gradual increase in its spatial aggregation. Grassland and unused land exhibited markedly higher LSI values than the other types and relatively stable trends over time, indicating comparatively complex patch shapes and boundaries. In contrast, cropland and construction land had lower LSI values, indicating relatively simpler patch configurations. Grassland and unused land exhibited the highest PLAND values throughout the study period, with only minor fluctuations, confirming their dominance in the landscape composition of the Qilian Mountains. The proportion of forestland and water bodies remained relatively stable, whereas construction land exhibited a sustained increase despite accounting for a low proportion of the total area, thus reflecting a gradual intensification of human activity in localized regions.

**FIGURE 4:**
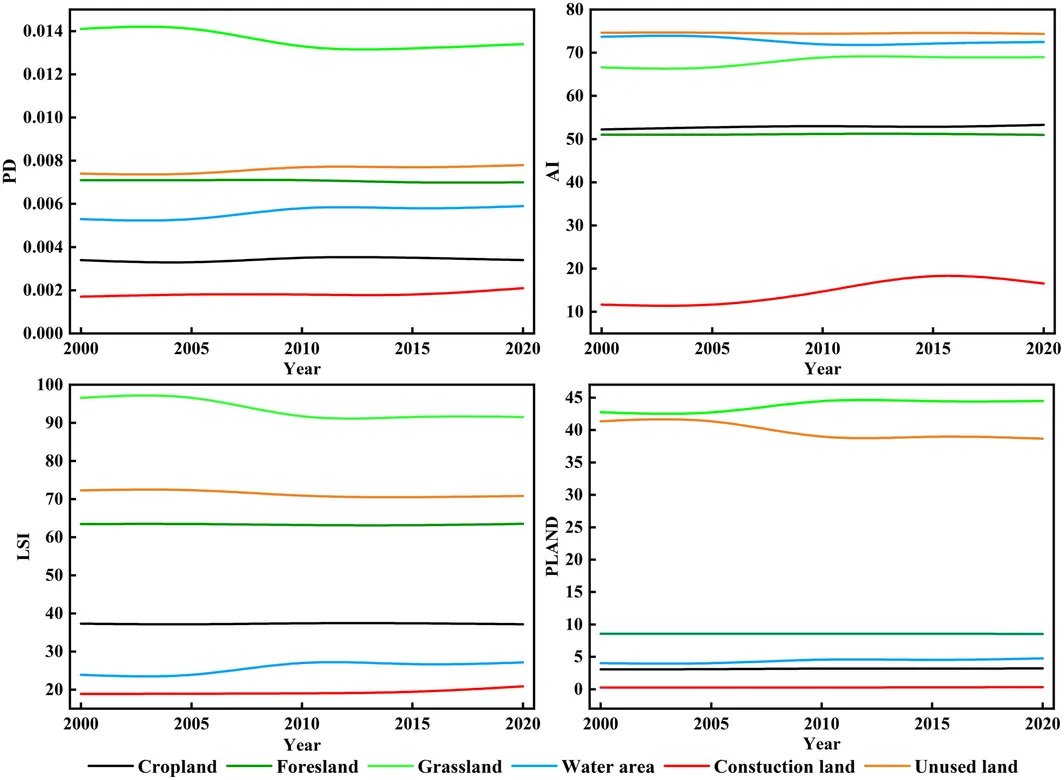
Temporal changes in landscape type indices.

### 3.3 Spatiotemporal Distribution of Landscape Pattern Indices

As shown in Figure 5, different landscape indices exhibited pronounced spatial heterogeneity and clear temporal evolution patterns throughout the study period. High‐value areas of PD were primarily located along the northeastern edge of the Qilian Mountains and the Hexi Corridor. In 2000, these high‐value areas were broadly and continuously distributed, whereas by 2020, their spatial extent had contracted, with low‐value areas gradually expanding into mid‐ to high‐altitude mountainous regions, indicating a spatial contraction of highly fragmented areas by the end of the study period.

**FIGURE 5:**
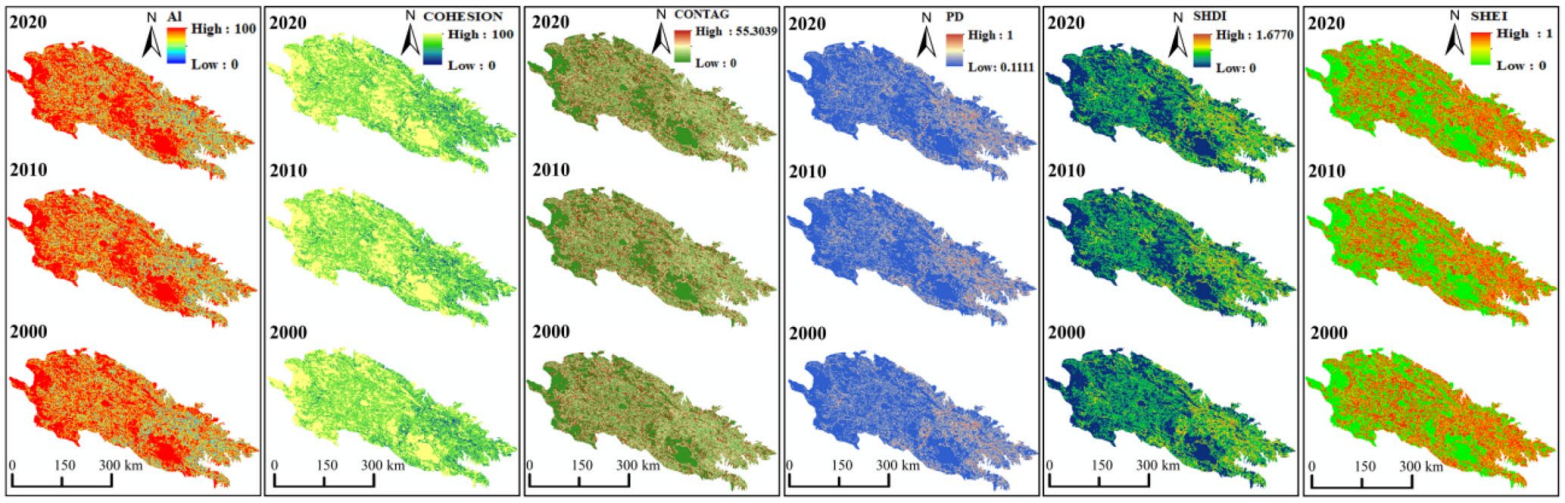
Spatial distribution of landscape pattern indices.

The spatial distribution patterns of CONTAG and COHESION were largely consistent, with high‐value areas concentrated in the midwestern and southern high‐altitude regions of the Qilian Mountains. From 2000 to 2020, these high‐value areas exhibited a continuous expansion, indicating an expansion of areas characterized by relatively high landscape contagion and connectivity. SHDI and SHEI displayed a clear east‐high, west‐low spatial pattern. High‐value areas were stably distributed in valley plains and piedmont transition zones and showed slight increases over the study period, indicating relatively high landscape diversity and evenness in these areas. The AI showed an overall upward trend, with high‐value areas evolving from scattered distributions toward more concentrated patterns. This reflects an increase in the spatial aggregation of patches in these areas over time.

### 3.4 Responses of Landscape Patterns to HAI

#### 3.4.1 Spatiotemporal Distribution of HAI

As shown in Figure 6, HAI in the Qilian Mountains during the study period exhibited pronounced spatial heterogeneity and stage‐specific temporal variations. Spatially, HAI displayed an overall pattern of higher values in the east and north and lower values in the west and south. High‐value areas were mainly concentrated along the southeastern and eastern margins of the Qilian Mountains, particularly along the Hexi Corridor and around major towns and transportation corridors, forming continuous or semi‐continuous high‐intensity clusters. In contrast, the midwestern and high‐altitude areas of the Qilian Mountains exhibited generally low HAI values, which were distributed over relatively large and contiguous areas.

**FIGURE 6:**
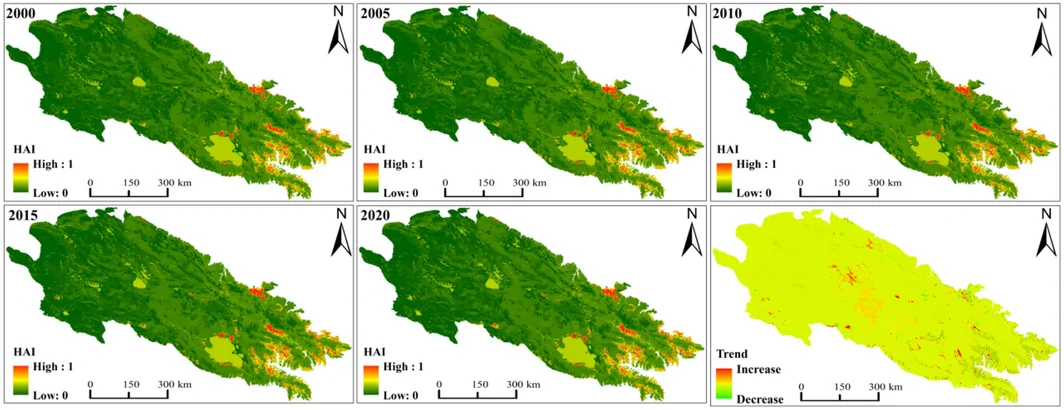
Spatial distribution of human activity intensity (HAI) in the Qilian Mountains.

Temporally, HAI remained relatively stable overall from 2000 to 2020 but exhibited a slight upward trend, with pronounced regional differences. Trend analysis indicated that most areas showed no significant changes and remained relatively stable throughout the study period. Areas with increasing HAI were mainly distributed around urban areas, irrigated agricultural areas, and along some transportation corridors, forming scattered or banded zones of increase. Conversely, some high‐altitude grasslands and forestlands exhibited slight decreases or remained relatively stable in HAI over the study period.

#### 3.4.2 Spatial Associations Between Landscape Pattern Indices and HAI

As shown in Figure 7, bivariate Moran's *I* and LISA analyses revealed distinct spatial associations between HAI and landscape pattern indices. At the global spatial level, HAI showed statistically significant negative spatial associations with AI and COHESION, with bivariate Moran's *I* values of −0.0863 and −0.1019, respectively (*p* = 0.001). These negative associations indicate that areas with relatively high HAI tended to be spatially associated with areas characterized by lower patch aggregation and connectivity. In contrast, HAI showed statistically significant positive spatial associations with PD, SHDI, and SHEI, with bivariate Moran's *I* values of 0.1083, 0.1198, and 0.0508, respectively (*p* = 0.001). This indicates that areas with relatively high HAI tended to be spatially associated with areas characterized by higher patch density, landscape diversity, and evenness.

**FIGURE 7:**
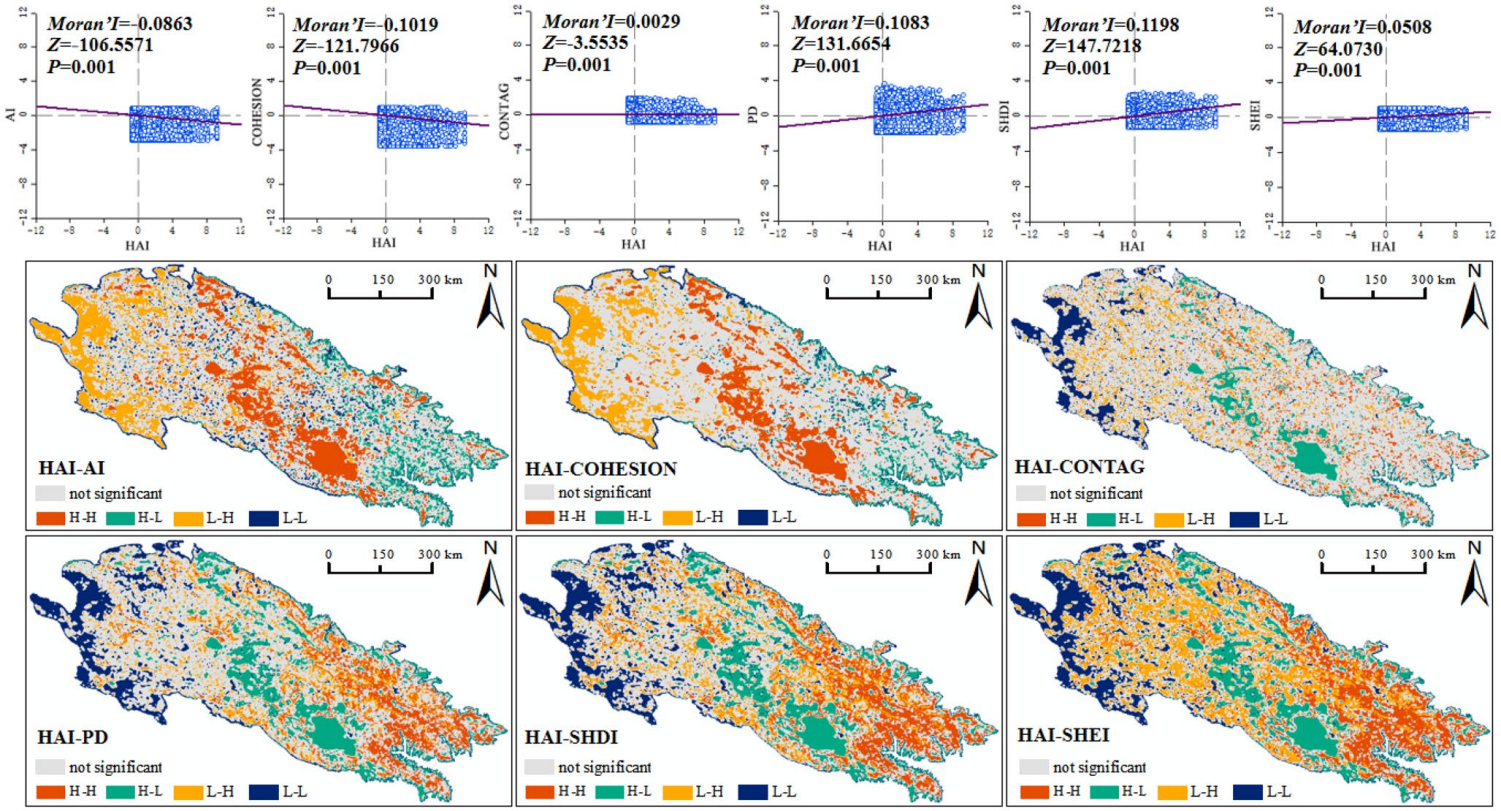
Cluster analysis and scatter plots of landscape pattern indices and human activity intensity (HAI).

At the local scale, the LISA results revealed pronounced regional differentiation. The eastern and northeastern margins of the Qilian Mountains were dominated by “high HAI‐high PD,” “high HAI‐high SHDI,” “high HAI‐high SHEI,” and “high HAI‐low AI/COHESION” clusters, indicating the spatial co‐occurrence of relatively high HAI with higher fragmentation and diversity but lower aggregation and connectivity. Conversely, the midwestern and high‐altitude regions predominantly exhibited “low HAI‐low PD,” “low HAI–low SHDI,” “low HAI–low SHEI,” “low HAI–low CONTAG,” and “low HAI‐high AI/COHESION” clusters, indicating that regions with low HAI were characterized by lower fragmentation, landscape diversity, evenness, and contagion, but higher aggregation and connectivity.

#### 3.4.3 Spatially Varying Relationships Between HAI and Landscape Pattern Indices

The GWR results revealed pronounced spatial heterogeneity in the relationships between HAI and the 6 landscape pattern indices across the Qilian Mountains (Figure 8). The local regression coefficients varied considerably in both magnitude and direction, indicating that the associations identified by the global spatial statistics were spatially non‐stationary. In general, the relationships of HAI with AI and COHESION exhibited similar spatial patterns. Negative coefficients (< 0) were extensively distributed in the western part of the study area and occurred sporadically in the central region, whereas the eastern part was dominated by relatively weak coefficients around zero, with locally positive associations. In contrast, the relationships of HAI with PD, SHDI, and SHEI showed broadly similar spatial configurations that differed markedly from those of AI and COHESION. Positive coefficients (> 0) were extensively distributed in the western region, with many areas exceeding 1.0, whereas negative coefficients (< 0) were concentrated mainly in the central part of the Qilian Mountains. Toward the eastern region, the coefficients were generally closer to zero and showed a mixture of weak positive and negative relationships. The broadly opposite coefficient patterns between AI/COHESION and PD/SHDI/SHEI indicate substantial spatial variation in the associations of HAI with landscape aggregation and connectivity versus patch density, diversity, and evenness. However, the relationship between HAI and CONTAG exhibited a more complex and spatially heterogeneous pattern. Positive and negative coefficients were interspersed across the study area, with relatively strong positive coefficients occurring mainly in parts of the western region and negative coefficients occurring discontinuously in the central and northern regions. Overall, the GWR results further demonstrated that the relationships between HAI and landscape patterns were highly spatially heterogeneous, with distinct regional differences among landscape aggregation, connectivity, fragmentation, diversity, and evenness.

**FIGURE 8:**
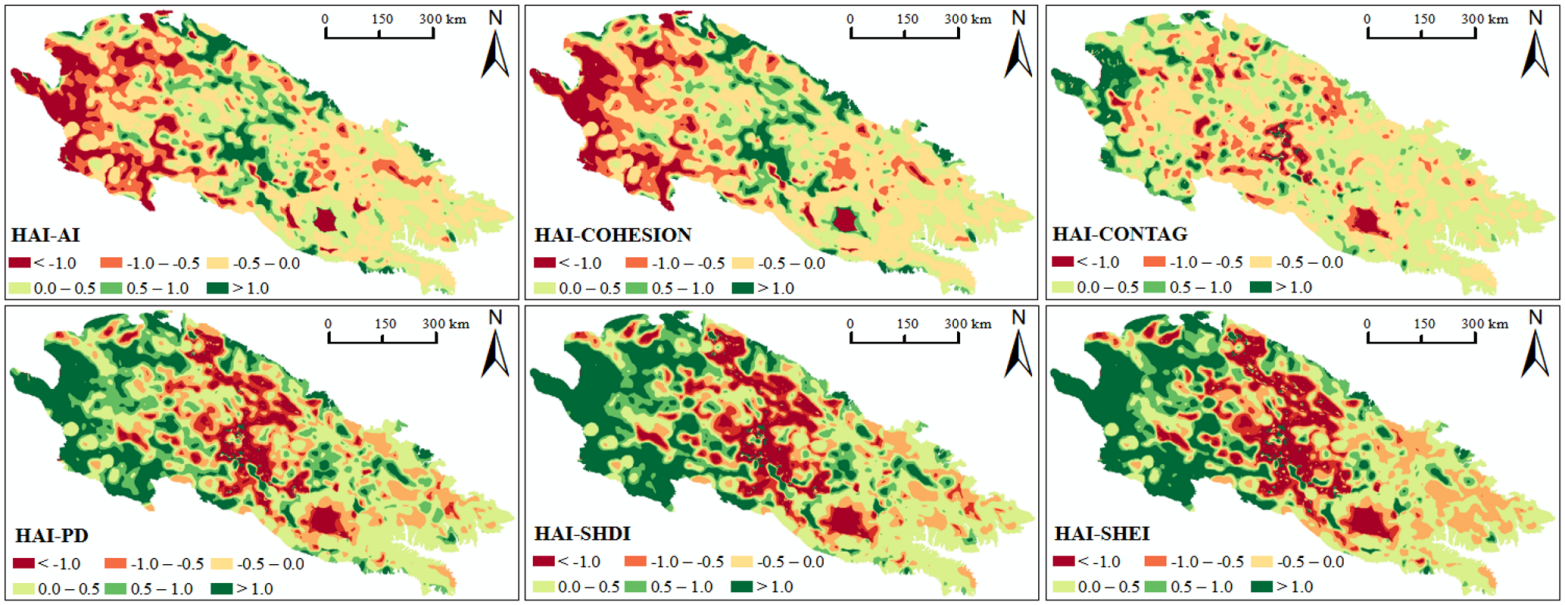
Spatial distribution of geographically weighted regression (GWR) coefficients between human activity intensity (HAI) and landscape pattern indices in the Qilian Mountains.

### 3.5 Spatiotemporal Distribution of LER

As shown in Figure 9, the LER in the Qilian Mountains exhibited pronounced spatial heterogeneity and temporal variation during 2000–2020. Spatially, LER generally increased from the southeastern to the northwestern parts of the Qilian Mountains. Low‐ and moderate‐low‐risk areas were extensively distributed in the eastern and southeastern regions, whereas moderate‐ and moderate‐high‐risk areas were more prevalent toward the western and northwestern parts of the study area. High‐risk areas occupied a relatively small proportion of the region and occurred mainly as localized patches. Despite some temporal fluctuations in individual risk classes, the overall spatial distribution of LER remained relatively stable throughout the study period. The LER trend analysis further revealed pronounced spatial heterogeneity in ecological risk evolution. Most areas within the study region exhibited no significant changes in LER, indicating that ecological risk remained relatively stable across much of the Qilian Mountains. Areas with increasing LER occurred mainly as scattered patches or small clusters in the northwestern and some central regions, whereas areas with decreasing LER were more continuously distributed in the eastern and northeastern regions.

**FIGURE 9:**
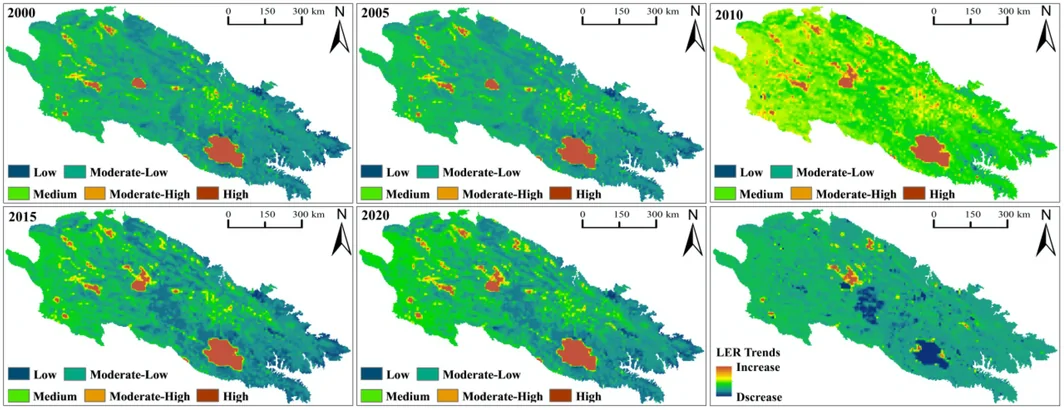
Spatial distribution of landscape ecological risk (LER).

Analysis of changes in the areas of different ecological risk levels (Figure 10) revealed distinct temporal trajectories during 2000–2020. The area of low‐risk zones remained relatively stable overall, with a slight decreasing trend. Moderate‐low‐risk areas decreased initially, subsequently increased markedly, and then declined and tended to stabilize. Moderate‐risk areas remained relatively stable during the early study period, increased markedly during the middle period, and subsequently decreased and gradually stabilized. Moderate‐high‐risk areas initially increased, reached a peak during the middle study period, and then gradually decreased. In contrast, the area of high‐risk zones exhibited relatively small variations throughout the study period, with only minor fluctuations over time. Overall, the five ecological risk levels exhibited different temporal trajectories, with more pronounced fluctuations occurring primarily in the moderate‐low, moderate, and moderate‐high risk categories.

**FIGURE 10:**
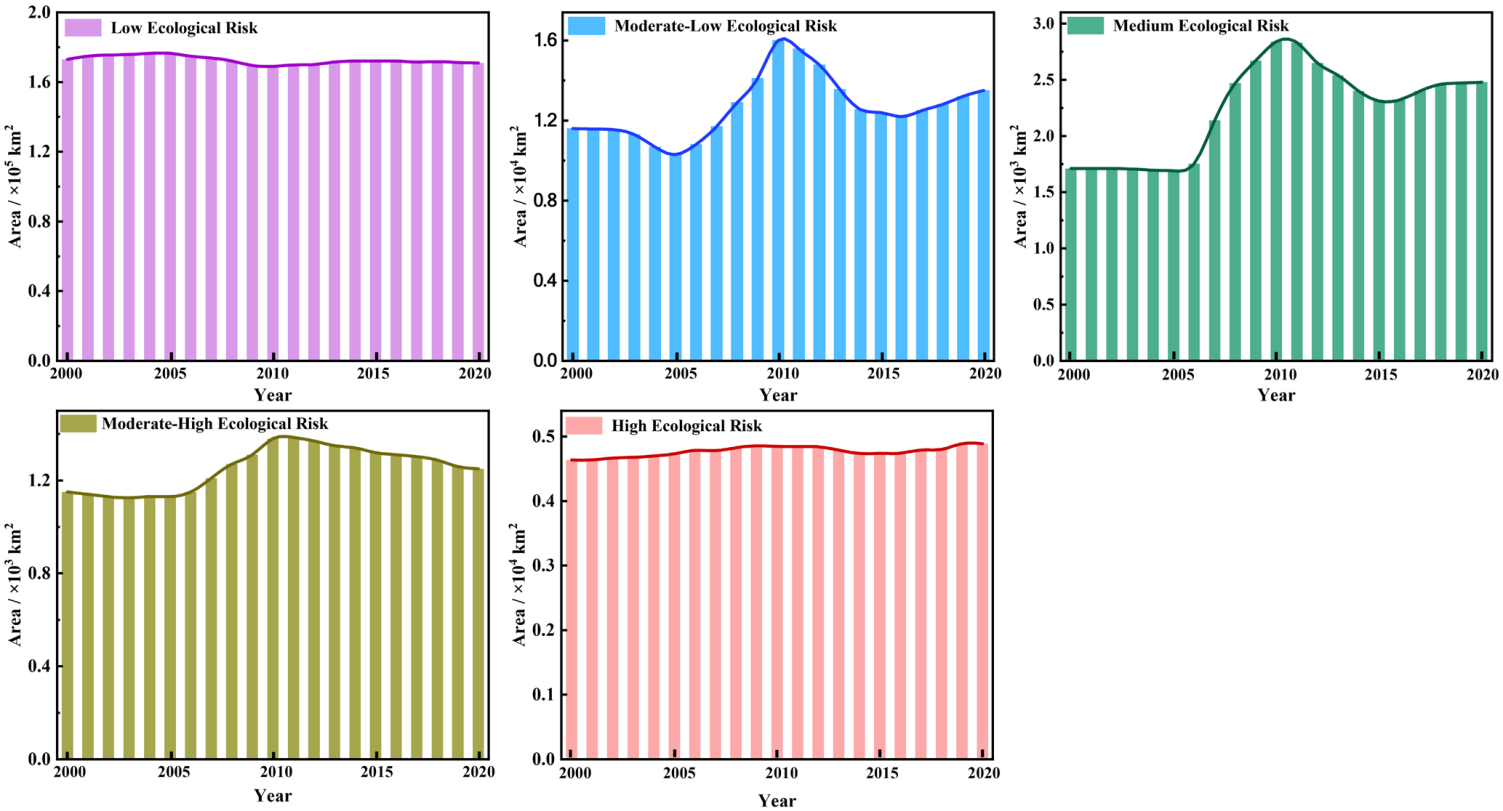
Trends in landscape ecological risk across different risk levels.

## 4 Discussion

### 4.1 Spatiotemporal Distribution and Coupling Characteristics of HAI and Landscape Patterns

The interactions between human activity and landscape patterns are important components of regional ecosystem structure and functional dynamics (Fu et al. 2023). Human activities such as land use changes, spatial expansion, and infrastructure development can modify landscape composition and configuration, with potential consequences for ecosystem stability and ecological security (Destoumieux‐Garzon et al. 2022; Dong et al. 2012). The spatiotemporal analyses of HAI and multidimensional landscape pattern indices in the Qilian Mountains from 2000 to 2020 performed in this study systematically revealed the spatial differentiation and coupling relationships between HAI and landscape pattern evolution in high‐altitude mountain ecosystems. Spatially, HAI in the Qilian Mountains remained generally low during the study period but exhibited pronounced heterogeneity, with a clear pattern of higher values in the east and lower values in the west. This distribution closely aligns with regional natural geographic conditions and socioeconomic development levels (Li, Feng, et al. 2021). The eastern and northeastern margins of the Qilian Mountains, which are adjacent to the Hexi Corridor, exhibit concentrated populations, agricultural development, and urban expansion; thus, human disturbance of the land surface is relatively strong (Xue et al. 2023). In contrast, the midwestern high‐altitude regions, which are characterized by rugged terrain, harsh climatic conditions, and strict protection in natural reserves and ecological function zones, exhibit low levels of HAI (Qian et al. 2021). Temporally, HAI exhibited relatively limited overall variation with a gradual upward trend from 2000 to 2020, although localized increases were evident in some areas.

The landscape patterns in the Qilian Mountains also exhibited pronounced spatiotemporal differentiation during the study period. Overall, the regional landscape structure remained relatively stable, although localized changes in fragmentation, aggregation, connectivity, and landscape diversity were observed. The eastern and northeastern regions generally exhibited relatively higher fragmentation and landscape heterogeneity, whereas the midwestern high‐altitude areas maintained stronger patch aggregation and connectivity. The partial spatial correspondence between these landscape characteristics and HAI suggests that human activities may be associated with regional differences in landscape configuration. However, the landscape structure is also strongly conditioned by the underlying topographic, climatic, and ecological characteristics of the Qilian Mountains (Qian et al. 2021).

Further spatial association analysis revealed significant spatial correlations between HAI and landscape pattern indices, suggesting that variations in human activity are closely associated with differences in landscape configuration (Xu et al. 2016). LISA clusters further confirmed the spatial heterogeneity of this association. In the northeastern Qilian Mountains, high‐HAI areas exhibited elevated patch density and landscape diversity as well as lower aggregation and connectivity, thus forming a typical “high‐disturbance–high‐fragmentation” landscape pattern. Conversely, in the midwestern high‐altitude regions, areas of low human activity corresponded to low patch density, high aggregation, and strong connectivity, reflecting a stable, naturally dominant landscape structure. The GWR results further revealed pronounced spatial non‐stationarity in these relationships (Li, Abuduwailia, et al. 2024; Li, Hu, et al. 2024). HAI showed broadly similar local relationships with AI and COHESION, whereas its relationships with PD, SHDI, and SHEI exhibited generally contrasting spatial patterns. Notably, the relatively weak global association between HAI and CONTAG masked substantial local variation, with both positive and negative relationships occurring across the study area. Together, these results indicate that the associations between human activity and landscape patterns in the Qilian Mountains are highly region‐dependent. On the one hand, natural geographic conditions such as rugged terrain and harsh climate constrain the spatial expansion of human activities, thereby indirectly shaping landscape spatial differentiation. On the other hand, socioeconomic development intensity and land use policies vary across regions, amplifying the spatial heterogeneity of the human activity‐landscape pattern. For example, in mid‐ to low‐altitude areas with favorable transportation and frequent land use transitions, landscape structure may be more strongly associated with human disturbances, whereas in high‐altitude ecological core zones, restricted human activity may contribute to the relatively intact landscape patterns.

From a temporal perspective, HAI and landscape patterns showed relatively gradual changes over the study period. Although the overall HAI in the Qilian Mountains exhibited limited changes, sustained land use adjustments and localized development gradually altered the landscape structural characteristics, with the eastern and northeastern regions showing particularly pronounced fragmentation trends (Xiang et al. 2021). The coexistence of a gradual increase in HAI and spatially heterogeneous changes in landscape patterns suggests that landscape responses to human activity are not spatially uniform. Overall, from 2000 to 2020, HAI and landscape pattern evolution in the Qilian Mountains demonstrated clear spatially heterogeneous associations, which may reflect the combined influence of natural constraints and anthropogenic disturbances. Terrain, climate, and ecological conservation conditions may regulate both the spatial distribution of human activities and their associations with landscape structure, thereby contributing to the observed spatial heterogeneity in landscape responses (Chang et al. 2025). These spatially heterogeneous relationships further reveal the interactions between human activities and landscape patterns in the Qilian Mountains and provide an important basis for interpreting the spatial variation in landscape ecological risk from a landscape structural perspective (Yang et al. 2022).

### 4.2 Relationships of HAI and Landscape Pattern Changes With LER

LER serves as a comprehensive indicator for assessing regional ecosystem structural stability, functional integrity, and responsiveness to natural and anthropogenic disturbances (Meng, Meng, et al. 2024; Lu et al. 2023). At its core, LER reveals the intrinsic linkages between changes in landscape patterns and the destabilization of ecological processes. The complex terrain, fragile ecological foundations, and high climate sensitivity of high‐altitude mountain ecosystems contribute to diverse and spatially heterogeneous mechanisms underlying ecological risk formation (Chang et al. 2025; Gao et al. 2021). This study systematically elucidated the evolution of regional ecological risk patterns based on the spatiotemporal distribution of LER in the Qilian Mountains from 2000 to 2020, thereby providing empirical support for understanding the ecological security framework of the northeastern Tibetan Plateau. From the overall distribution pattern, low and moderate‐low ecological risk zones dominated the study region. This pattern indicates that the ecosystem stability of the Qilian Mountains, which represents a critical ecological security barrier, has largely been maintained over the study period. These results are consistent with the region's long‐standing status as a predominantly natural ecosystem with limited development intensity. Meanwhile, long‐term ecological protection, including the establishment and management of nature reserves and ecological function zones, may also have contributed to limiting large‐scale land‐use transformation and maintaining relatively stable landscape structures (Li, Feng, et al. 2021; Liu, Si, et al. 2024). The overall stability of landscape patterns during the study period was therefore consistent with the predominance of low ecological risk.

Despite the overall low level of ecological risk, LER in the Qilian Mountains exhibited pronounced spatial heterogeneity, which was manifested as distinct regional differentiation. A clear spatial gradient in ecological risk was observed, with a general increase from the southeast toward northwest and from low to high altitudes. This spatial differentiation reflects the important role of natural environmental gradients in ecological risk formation (Li et al. 2023). The northeastern Qilian Mountains feature relatively gentle terrain, favorable hydrothermal conditions, and high vegetation cover, which collectively confer relatively high ecosystem structural stability (Qian et al. 2021). In contrast, the midwestern high‐altitude mountains are constrained by steep topography, frequent freeze–thaw cycles, and poorly developed soils, resulting in reduced ecosystem resistance to disturbances and a higher propensity for elevated ecological risk (Liu et al. 2025). Temporally, LER in the Qilian Mountains displayed a pattern of “overall stability with local fluctuations” from 2000 to 2020. Low‐risk areas remained relatively stable with a slight decreasing trend, whereas moderate‐low‐, moderate‐, and moderate‐high‐risk areas exhibited varying degrees of temporal fluctuations, including episodic expansion and subsequent contraction. This evolution suggests that changes in regional ecological risk are not unidirectional or linear but reflect complex dynamic adjustments associated with both natural environmental variation and human disturbances (Lin et al. 2025). Notably, in some high‐altitude and ecological transition zones, risk levels shifted from moderate to relatively high, highlighting the potential vulnerability of alpine ecosystems under the context of ongoing climate change (Xia et al. 2021).

From a driving‐mechanism perspective, the spatiotemporal distribution of LER in the Qilian Mountains may reflect the combined influence of multiple natural processes and anthropogenic factors (Li, Zhou, et al. 2021). First, climate change constitutes a critical background factor influencing regional ecological risk dynamics (Qi et al. 2022). In recent years, the region has experienced a pronounced warming trend and increased interannual precipitation variability, which may intensify freeze–thaw erosion, soil moisture imbalances, and vegetation degradation risks (Gao et al. 2021). In high‐altitude areas, widespread permafrost and seasonally frozen ground are highly sensitive to warming. Moreover, permafrost degradation and active‐layer deepening can affect soil stability, surface hydrological processes, and vegetation conditions, potentially increasing the vulnerability of alpine landscapes (Tas et al. 2018). These climate‐related cryospheric processes may partly explain the occurrence of relatively high ecological risk in some high‐altitude areas where direct human activity remains limited. Second, the complex topography may influence ecological risk by modulating water–heat redistribution and landscape connectivity (Gunderson 2023). Steep slopes and highly fragmented terrain can constrain vegetation recovery and may contribute to the persistence of high‐risk patches in specific areas (Kindermann et al. 2025). Although the overall HAI in the Qilian Mountains remains low, localized development may also contribute to spatial variations in LER. Changes in ecological risk levels in some areas may be associated with grassland management, legacy impacts of mineral resource extraction, and infrastructure development (Jia et al. 2022; Tsai and Lee 2023). Therefore, the observed LER pattern is more appropriately interpreted as the combined outcome of natural environmental vulnerability and spatially heterogeneous anthropogenic disturbance rather than as a direct response to human activity alone.

Notably, the gradual increase in HAI during 2000–2020 coexisted with an overall stable LER pattern and localized decreases in moderate‐ and high‐risk areas, indicating that changes in HAI and LER do not necessarily follow a simple linear relationship. Although HAI increased gradually at the regional scale, this increase was spatially heterogeneous rather than uniformly distributed across the Qilian Mountains. Moreover, as shown by the spatial autocorrelation and GWR analyses, the relationships between HAI and landscape pattern indices varied substantially among regions, suggesting that similar changes in human activity may correspond to different landscape responses under different environmental settings. Therefore, an increase in regional HAI does not necessarily translate directly into a proportional increase in landscape ecological risk. Meanwhile, in some middle‐ and high‐altitude areas where human activities are limited, variations in LER may be more strongly associated with natural environmental conditions, such as climate change and freeze–thaw erosion, which may further weaken the spatial correspondence between HAI and LER (Lin et al. 2025).

The localized reduction in ecological risk after 2010 also coincided with the continued implementation of ecological protection and restoration measures in the Qilian Mountains, including grazing restrictions, rotational grazing, degraded grassland restoration, and ecological compensation programs (Zhang et al. 2024). These measures may have contributed to vegetation recovery and the maintenance or improvement of landscape conditions in some areas, potentially offsetting part of the ecological pressure associated with increasing human activity. However, because the present study did not directly quantify policy effects, the localized decline in ecological risk cannot be attributed solely to ecological protection measures. Rather, the coexistence of increasing HAI and locally decreasing LER likely reflects the combined effects of the spatial concentration of human activities, heterogeneous landscape responses, ecological restoration, and natural environmental processes (Liu et al. 2025).

### 4.3 Implications for Adaptive Ecosystem Management and Sustainable Development

Mountain ecosystems occupy an irreplaceable strategic position in the global ecological security framework. They serve not only as critical refuges for biodiversity but also as essential regulators of water resources, climate, and regional ecological stability. The pronounced spatial heterogeneity in HAI, landscape patterns, and LER identified in this study indicates that ecosystem management in the Qilian Mountains should move beyond generalized protection toward spatially differentiated and adaptive strategies. Management priorities should be tailored to regional differences in human disturbance, landscape structure, ecological risk, and dominant ecological functions, while also considering potential future changes in climate‐sensitive alpine ecosystems to support more proactive risk prevention.

First, management of areas with relatively intensive human activities should focus on limiting further landscape fragmentation. In the eastern and northeastern middle‐ and low‐altitude areas, where relatively high HAI is associated with greater landscape fragmentation, further expansion and spatial dispersion of construction land should be carefully controlled, landscape segmentation associated with infrastructure development should be minimized, and the continuity of key ecological patches and corridors should be maintained. Monitoring in these areas should focus on land‐use conversion, patch fragmentation, and landscape connectivity to identify emerging ecological risks associated with changes in landscape structure.

Second, differentiated measures should be adopted according to ecological vulnerability and dominant ecosystem functions. In the central‐western and middle‐ to high‐altitude areas characterized by relatively high ecological risk but limited human activity, priority should be given to conserving naturally fragile alpine ecosystems, minimizing additional anthropogenic disturbance, and monitoring vegetation, permafrost, and freeze–thaw processes. In key water‐conservation and headwater areas, protection of alpine grasslands, wetlands, and riparian ecosystems should be strengthened, while land‐use changes that may impair water‐retention and hydrological regulation functions should be restricted (Guo et al. 2024). In existing and historical mineral development areas, particularly those overlapping with ecologically sensitive or relatively high‐risk landscapes, new disturbances should be strictly controlled and restoration of abandoned or degraded mining land should be prioritized (Yang et al. 2024). In low‐risk areas with relatively stable landscape structures, maintaining existing ecological conditions and avoiding excessive intervention should remain the primary management objective.

Finally, sustainable development in the Qilian Mountains should balance necessary human activities with the protection of ecologically vulnerable areas rather than relying on the complete exclusion of human activities. Development activities should be spatially guided away from high‐risk and ecologically sensitive areas, while future climate and land‐use change scenarios should be incorporated into ecological risk assessment and spatial planning to project potential changes in ecological risk and identify areas where preventive interventions may be required. In high‐altitude climate‐sensitive areas, the long‐term monitoring of vegetation dynamics, permafrost conditions, and freeze–thaw processes should be integrated with HAI and landscape‐pattern monitoring to help distinguish the effects of natural environmental changes from those of anthropogenic pressures and support adaptive management (Sun et al. 2022). Future assessments integrating climate, cryospheric, vegetation, and land‐use scenarios could further support the early identification of emerging ecological risk hotspots and the timely adjustment of conservation and restoration priorities. Such spatially differentiated, forward‐looking, and adaptive management can contribute to enhancing ecosystem resilience while promoting long‐term coordination between ecological conservation and regional sustainable development.

## 5 Conclusions

Based on land use data from 2000 to 2020, this study constructed an integrated framework that combines the HAI indicator, multi‐level landscape pattern metrics, and an LER assessment model to systematically investigate the spatiotemporal dynamics and spatial relationships among human activities, landscape pattern evolution, and ecological risk in the Qilian Mountains. Using spatial statistical, spatial autocorrelation, and geographically weighted regression (GWR) analyses, this study identified the spatial associations and spatially varying relationships between human activities and landscape patterns and examined their relationships with ecological risk, thereby providing comprehensive evidence for ecological risk identification and management in high‐altitude mountain systems. The main conclusions are as follows:

1. During the study period, the overall HAI in the Qilian Mountains remained relatively low but exhibited pronounced spatial heterogeneity, which was characterized by a clear decreasing trend from east to west and from low to high elevations. Areas with relatively high HAI were primarily concentrated in the eastern and southeastern regions, particularly around urban centers and adjacent valleys, whereas areas with low HAI were primarily distributed in the central‐western and high‐altitude areas. Temporally, HAI showed a gradual increase with relatively limited temporal variation from 2000 to 2020, while its spatial pattern remained generally stable.
2. The overall landscape pattern of the Qilian Mountains remained generally stable throughout the study period; however, localized increases in landscape fragmentation and pronounced spatial differences in connectivity and aggregation were observed. Landscape metrics exhibited significant spatially heterogeneous relationships with HAI. Areas with higher HAI were typically associated with increased patch density and reduced aggregation and connectivity, whereas regions with low anthropogenic disturbance generally exhibited lower fragmentation and higher aggregation and connectivity. The GWR results further revealed pronounced spatial non‐stationarity in these relationships. HAI showed broadly similar local relationships with AI and COHESION, whereas its relationships with PD, SHDI, and SHEI exhibited generally contrasting spatial patterns.
3. LER in the Qilian Mountains was dominated by low‐ and moderate‐low‐risk levels, indicating a generally low landscape ecological risk and a relatively stable spatial risk pattern across the region. Nevertheless, clusters of high‐risk patches were evident in the central‐western and high‐altitude areas, with ecological risk displaying an increasing spatial trend from the southeast to northwest. The spatial distribution of LER did not correspond directly to that of HAI, as some middle‐ and high‐altitude areas exhibited relatively high ecological risk despite limited human activity. This spatial mismatch highlights the complexity of ecological risk patterns in high‐altitude mountain environments.

Overall, by integrating the assessment of HAI, landscape pattern dynamics, and ecological risk within a unified analytical framework, this study provides a more comprehensive understanding of the spatial relationships among human activity, landscape structure, and ecological risk in high‐altitude mountainous regions.

## Author Contributions

**Shengji Jiang:** conceptualization (lead), data curation (lead), formal analysis (equal), methodology (equal), resources (supporting), software (lead), writing – original draft (lead). **Heng Ren:** methodology (supporting), resources (equal), supervision (lead), writing – review and editing (lead). **Hua Xu:** investigation (equal), project administration (supporting), validation (equal). **Tao Zhou:** data curation (equal), software (supporting), validation (supporting).

## Funding

This work was supported by the National Social Science Foundation of China (no. 23XGL035), Key Research and Development Program of the Xizang Autonomous Region (No. XZ202601ZY0054) and Western Light Program of the Chinese Academy of Sciences (No. E5220901).

## Ethics Statement

This article does not contain any studies with human participants or animals performed by any of the authors.

## Conflicts of Interest

The authors declare no conflicts of interest.

## Supporting information


**File S1:** Supporting data for landscape pattern indices and landscape ecological risk in the Qilian Mountains.

## Data Availability

All the required data are uploaded as Supporting Information.
